# Effect of *Callistemon citrinus* Phytosomes on Oxidative Stress in the Brains of Rats Fed a High-Fat–Fructose Diet

**DOI:** 10.3390/biom15081129

**Published:** 2025-08-05

**Authors:** Oliver Rafid Magaña-Rodríguez, Luis Gerardo Ortega-Pérez, Aram Josué García-Calderón, Luis Alberto Ayala-Ruiz, Jonathan Saúl Piñón-Simental, Asdrubal Aguilera-Méndez, Daniel Godínez-Hernández, Patricia Rios-Chavez

**Affiliations:** 1 Facultad de Biología, Universidad Michoacana de San Nicolás de Hidalgo, Morelia 58030, Mexico; oliver.rodriguez@umich.mx (O.R.M.-R.); gerardo.ortega@umich.mx (L.G.O.-P.); 1208935x@umich.mx (A.J.G.-C.); 1232816g@umich.mx (L.A.A.-R.);; 2 Instituto de Investigaciones Químico Biológicas, Universidad Michoacana de San Nicolás de Hidalgo, Morelia 58030, Mexico; amendez@umich.mx (A.A.-M.); daniel.godinez@umich.mx (D.G.-H.)

**Keywords:** antioxidant, brain, *Callistemon citrinus*, oxidative stress, phytosomes

## Abstract

*Callistemon citrinus* has shown antioxidant and anti-inflammatory properties in certain tissues. However, its impact on the brain remains unproven. This study investigates the effect of *C. citrinus* extract and phytosomes on the oxidative status of the brains of rats fed a high-fat–fructose diet (HFD). Fifty-four male Wistar rats were randomly divided into nine groups (*n* = 6). Groups 1, 2, and 3 received a standard chow diet; Group 2 also received the vehicle, and Group 3 was supplemented with *C. citrinus* extract (200 mg/kg). Groups 4, 5, 6, 7, 8, and 9 received a high-fat diet (HFD). Additionally, groups 5, 6, 7, 8, and 9 were supplemented with orlistat at 5 mg/kg, *C. citrinus* extract at 200 mg/kg, and phytosomes loaded with *C. citrinus* at doses of 50, 100, and 200 mg/kg, respectively. Administration was oral for 16 weeks. Antioxidant enzymes, biomarkers of oxidative stress, and fatty acid content in the brain were determined. A parallel artificial membrane permeability assay (PAMPA) was employed to identify compounds that can cross the intestinal and blood–brain barriers. The HFD group (group 4) increased body weight and adipose tissue, unlike the other groups. The brain fatty acid profile showed slight variations in all of the groups. On the other hand, group 4 showed a decrease in the activities of antioxidant enzymes SOD, CAT, and PON. It reduced GSH level, while increasing GPx activity as well as MDA, 4-HNE, and AOPP levels. *C. citrinus* extract and phytosomes restore the antioxidant enzyme activities and mitigate oxidative stress in the brain. *C. citrinus* modulates oxidative stress in brain tissue through 1.8-cineole and α-terpineol, which possess antioxidant and anti-inflammatory properties.

## 1. Introduction

From 1990 to 2021, the number of men and women with overweight and obesity increased by 1.0 billion and 1.1 billion, respectively. It is projected that by 2050, 3.8 billion people will have this condition pathology [1].

Obesity and excessive fat accumulation in the body significantly generate reactive oxygen species (ROS). A decrease in antioxidant defenses and an increase in ROS indicate a state of oxidative stress that damages biomolecules such as carbohydrates, lipids, proteins, and nucleic acids [2].

High-fat, high-sugar diets are widely consumed worldwide, especially in the United States. This diet encourages an increase in reactive oxygen species and free radicals, ultimately leading to a state of oxidative stress [3]. The harmful effects of a high-fat diet (HFD) on cognitive function are mainly caused by increased adiposity and the subsequent pro-inflammatory and pro-oxidative impact on the brain [4]. These effects result from decreased activity of antioxidant enzymes such as superoxide dismutase (SOD), catalase (CAT), glutathione peroxidase (GPx), and paraoxonase-1 (PON-1) [5]. Additionally, HFD increases the levels of lipoperoxidation products in the brain (MDA and 4-HNE), which leads to protein damage [6,7].

Previous studies have demonstrated that the types of fatty acids in the diet play a key role in the pathophysiological changes observed in rodent models [8]. Pistell et al. [9] showed that a diet high in saturated fatty acids for rodents increases brain inflammatory markers. Similarly, Chen et al. [10] reported that mice fed a high-fat diet for 24 weeks showed increased expression of brain senescence markers. A high-fat diet also caused mitochondrial changes in microglial cells [11]. Conversely, consuming a high-sucrose or high-fructose diet triggers inflammation in the hippocampus, leading to memory impairments [12].

Orlistat (Xenical) is a lipase inhibitor approved by the FDA (Food and Drug Administration) for the treatment of obesity; however, it has also been reported to cause adverse effects, including difficulty controlling bowel movements, steatorrhea, flatulence, diarrhea, and stomach pain [13]. Natural products are widely used due to their diverse compounds, which can produce beneficial effects and often cause fewer side effects. Nonetheless, they must be present in high concentrations to achieve efficacy.

Phytosomes, also known as herbosomes, represent a novel technology utilizing vesicular systems for drug delivery. They consist of a phytoactive or natural product encapsulated within a phospholipid membrane, forming lipid-based nanoparticles that enhance the absorption and bioavailability of soluble compound drugs [14]. The lipid nature of the cell membrane improves its permeability and shields bioactive compounds from degradation in the gastrointestinal tract [15].

*Callistemon citrinus* belongs to the Myrtaceae family, is native to Australia, and was used by the Aboriginal people for the treatment of diarrhea, rheumatism, and cough. The first studies utilized leaves and flowers, reporting their antibacterial and antifungal properties [16]. *C. citrinus* has also been associated with anti-inflammatory, elastase inhibitory, molluscicidal, cardioprotective, antioxidant, and protective effects against type 2 diabetes [17]. Lopez-Mejia et al. reported the potential of *C. citrinus* leaf extract to inhibit colon cancer [18]. Piñón-Simental et al. found that the extract has a protective effect on the gastric mucosa against indomethacin [19]. Recently, we demonstrated that a high-fat, fructose-rich diet causes obesity and oxidative stress in the liver, heart, kidneys, and stomach of rats fed for 15 weeks [20]. Conversely, administering phytosomes loaded with *C. citrinus* leaf extract reduces oxidative stress and demonstrates anti-obesity, antioxidant, and anti-inflammatory effects [21].

We previously reported the phytochemical composition of the terpenes in *Callistemon citrinus* using gas chromatography–mass spectrometry (GC-MS) and two-dimensional gas chromatography (GC × GC) [22]. Additionally, a toxicology study conducted by Ortega-Pérez et al. [20] found that neither acute nor sub-chronic exposure exhibited toxicity. Finally, Ortega-Pérez et al. [23] identified p-coumaric acid, gallic acid, and ellagic acid in *C. citrinus* and characterized the phytosomes loaded with *C. citrinus*. The biological activity of *C. citrinus* is mainly attributed to the phenolic and terpenoid compounds previously reported, including several bioactive substances such as 1,8-cineole, limonene, α-terpineol, and terpinolene [24]. Numerous studies have investigated the role of these compounds as antioxidant and antiradical agents in mitigating oxidative stress caused by brain damage [25]. The *C. citrinus* phytosomes have been proven to be more stable and soluble than *C. citrinus* extract. This formulation enhances the absorption of polar compounds and creates a hydrophobic bilayer for non-polar substances. This contrasts with the low bioavailability and absorption of terpenes, phenolic, and flavonoid compounds when used orally. The use of *C. citrinus* extract and phytosomes has the advantage of containing compounds that protect the brain from oxidative stress; however, this has not been studied. On the other hand, Rule et al. [26] reported the effect of diet on the fatty acid profile in the brain. Additionally, Ulmann et al. [27] showed that the concentration of arachidonic acid decreases in brain phosphatidylinositol and phosphatidylserine with age. This study investigates the impact of *C. citrinus* extract and phytosomes loaded with *C. citrinus* on oxidative stress in the brains of rats fed a high-fat–fructose diet and determines whether dietary fat–sugar induced differences in the brain fatty acids.

## 2. Materials and Methods

### 2.1. Chemical Materials

Boron trifluoride, Bradford reagent, dodecane, ferrous ammonium sulfate, o-dianisidine dihydrochloride, potassium iodide, 1-methyl-2-phenylindole, methanesulfonic acid, 5,5′-dithiobis 2-nitrobenzoic acid, reduced glutathione, cumene hydroperoxide, sodium carbonate, riboflavin, 4-nitrophenylacetate, and all other chemicals and solvents were purchased from Sigma-Aldrich (Carlsbad, CA, USA) (Merck/Mexico/Life Science Products & Service Solutions).

### 2.2. Biological Material and Preparation of Ethanolic Extract

Leaves from *Callistemon citrinus* plants were collected in Morelia, Michoacán, Mexico. Fresh leaves were crushed and macerated in ethanol 96% at a 1:10 ratio (*w*/*v*). The ethanolic extract was stored in the dark at 4 °C for 7 days. The leaf extract was dried using a rotary evaporator with a vacuum pump at 45 °C. The concentrated extract was stored in a refrigerator at 4 °C until further use. The Supplementary File S1 shows the chemical composition of *Callistemoon citrinus* and the chromatogram.

### 2.3. Preparation of Phytosomes

The preparation of phytosomes followed the protocol outlined by Ortega-Pérez et al. [23]. The mixture included 50 mL of hydration medium (0.01 M phosphate buffer solution, 150 mM NaCl, pH 7.4), 1:1 (*w*/*w*) of soybean phosphatidylcholine and *C. citrinus* pure extract, 1 g of *C. citrinus* extract, and 1 g of soybean phosphatidylcholine to achieve a final concentration of 200 mg/mL; 0.5 g of *C. citrinus* extract and 0.5 g of soybean phosphatidylcholine for a final concentration of 100 mg/mL; and 0.25 g of *C. citrinus* extract and 0.25 g of soybean phosphatidylcholine for a final concentration of 50 mg/mL—all in the same volume of hydration medium. Additionally, 0.72 g of Tween 80 and 1% ethyl acetate were added to improve solubility. The mixture was then emulsified using an ultrasonicator (VCX 500, SONICS, Oklahoma City, OK, USA) at 25% amplitude for 10 min at 10 °C. This phytosomal complex was stored in an amber-colored glass bottle at 20 °C.

#### 2.3.1. Lyophilization and Scanning Electron Microscopy (SEM)

Phytosome samples were frozen at −80 °C for 8 h, then lyophilized in a high-vacuum environment of 34 Pa using a lyophilizer (Labconco Plus 12; Labconco, Kansas City, MO, USA) for 8 h with a condenser set at −43 °C. Lyophilized phytosomes were stored in a sealed glass ampoule at 4 °C. One drop of the lyophilized sample was placed on a brass electron microscope stub and coated with copper particles for sputtering. Representative images of the samples were taken, and particle diameters were calculated using scanning electron microscopy (JEOL JSM-7600F SEM, JEOL, Akishima, Japan) at an accelerating voltage of 20.0 kV and a working distance of 15.1 mm. Details of the morphological structure of the phytosomes were observed at an amplitude of up to 10,000× and a working distance that allowed for detailed observations at increasing depths of focus.

#### 2.3.2. Particle Size and Polydispersity Index

The mean particle size and polydispersity index (PDI) (which refers to the width of a particle size distribution) were measured using a Nano Particle Analyzer SZ-100 (Horiba, Kyoto, Japan) based on the principle of dynamic light scattering; Ludox TM silica served as the reference material [23]. Ludox TM-50 was diluted to 10% with 0.01 M KCl. Approximately 10 milliliters of KCl/LUDOX solution were filtered through a 2.5 µm filter. The samples were equilibrated for 2 min at 25 °C and then placed in a plastic cuvette for analysis at 90° scattering angles. All batches were performed in triplicate, and the mean and SD were calculated.

#### 2.3.3. Entrapment Efficiency

The entrapment efficiency of *C. citrinus* phytosomes was determined using a UV–visible spectrophotometer [23]. Approximately 1 mL of dialyzed vesicular suspension was taken and diluted with 0.1 mL of Triton X-100. The solution was centrifuged at 1350× *g* for 5 min, and the supernatant was diluted with ethanol. The amount of drug entrapped was analyzed spectrophotometrically at a maximum wavelength of 425 nm against ethanol containing Triton X-100 as a blank. Equation (1) computes the efficiency of entrapment (EE); Tdrug is the total amount of drug; Edrug is the extract entrapment in the formulation (phytosome); and Udrug is the extract not entrapped in phytosomal formulation.

(1) $$EE=\frac{Edrug}{Tdrug}\times 100\%=\frac{Edrug}{Edrug+Udrug}\times 100\%=\left(1-\frac{Udrug}{Edrug+Udrug}\right)\times 100\%$$

#### 2.3.4. Stability and Solubility

The stability analysis was conducted by storing the phytosomes at 20 ± 2 °C and 4 ± 1 °C, and the particle size was measured on days 1, 3, 5, and 10 after storage. It was then measured again after three and a half months.

Solubility analysis was performed by dissolving 2 mg of each formed complex (soybean phospholipid particles) and *C. citrinus* leaf extract in 5 mL of different solvents within small volumetric flasks. The solutions were stirred continuously for 1 h [23]. The experiments were conducted in triplicate.

### 2.4. Animals

White male Wistar rats were bred and maintained in a cage in the laboratory of the Institute of Chemical-Biological Research of UMSNH under bioterium conditions: a 12 h light/dark cycle, relative humidity of 60–70%, an average temperature of 20 °C, and food and water provided ad libitum. Young adults, aged 2 months and weighing between 200 and 250 g, were used in all treatments. The experimental process followed the guidelines for the care and use of laboratory animals as outlined in the Mexican Official Standard (NOM-062-ZOO-1999) [28] by the Mexican Secretary of Agriculture, Livestock, Rural Development, Fishing, and Food (published in the Official Gazette, Mexico City 2001). The Animal Bioethical Committee of UMSNH approved the study on 12 January 2024, with approval code ID IIQB-CIBE-06-2024. All efforts were made to minimize animal suffering and reduce the number of animals used in the study. The use of Wistar rats instead of other strains is based on their ease of handling, genetic uniformity, and ability to produce reproducible results.

### 2.5. Experimental Design, High-Fat–Fructose Diet

The model was conducted using a high-fat–fructose diet, following the protocol of Ortega-Pérez et al. [20]. Rats were given a diet consisting of 45% Rodent Diet^®^ brand rat food, which provided 0.20 g of protein, 0.77 g of carbohydrates, 0.03 g of fat, 0.12 g of calcium, 0.012 g of phosphate, 0.021 g of sodium, and 0.007 g of potassium per gram, supplying 3.3 calories per gram. The HFD also included 14.8% pork lard and 14.8% vegetable shortening (INCA brand), and the food was supplemented with 25% fructose. The detailed fatty acid composition is presented in Table 1. All ingredients were mixed and formed into pellets for use; HFD was prepared daily to avoid oxidation. Table S2 shows the composition of the control diet (see Supplement Material). The animals were randomly divided into nine groups (*n* = 6 per group): Group 1 was given a chow diet and a saline solution (C); Group 2 followed a chow diet and the vehicle (C + V); Group 3 was provided a chow diet with *C. citrinus* leaf extract (200 mg/kg) (C + C.c); Group 4 received an HFD and a saline solution; Group 5 received an HFD with orlistat (5 mg/kg) (HFD + Orl); Group 6 received an HFD with *C. citrinus* leaf extract (200 mg/kg) (HFD + C.c); Groups 7, 8, and 9 received HFD with *C. citrinus* phytosomes at 50, 100, and 200 mg/kg, respectively (HFD + P50; P100 and P200). The dosage of phytosomes was chosen based on a previous study [23].

Treatments were given once daily by oral gavage at 7:00 a.m. throughout the 16 weeks. The weights of all groups were measured weekly to monitor weight gain throughout the study. The percentage of total weight gain (X) was calculated as X = [(Final BW − initial BW)/Initial BW × 100]. In addition to weight gain, the Lee index and adiposity index were also calculated to demonstrate the animals’ obesity. The Lee index (g/cm), which is equivalent to BMI, was calculated as LI = (3√BW)/NAL × 10, and the equation to assess the percentage of body fat (%) is 0.73 (LI-280.8). In this way, the fat percentage depends only on the weight and the nasal–anus length of the rats. The adiposity index (AI) was calculated as AI = (total adipose tissue weight/final BW) × 100 [23] (Table 2). At the end of the experiment, the animals were anesthetized with an intraperitoneal injection of sodium pentobarbital (50 mg/kg). The entire brain was collected, washed with a 0.9% saline solution, weighed, and stored at −80 °C for further analysis.

### 2.6. Preparation of Brain Tissue Homogenates

The whole brain was thoroughly homogenized on ice at a ratio of 1:4 (*w*/*v*) with 10 mM phosphate buffer (pH 7.4) in a mortar until the tissue was disintegrated. After that, it was centrifuged at 13,000 rpm for 20 min at 4 °C, and the supernatant was collected and stored at −20 °C. Total proteins were measured as follows: 1 μL of brain homogenate was mixed with 799 μL of distilled water, and then 1 mL of Bradford reagent was added. The mixture was incubated at room temperature for 5 min, and the absorbance was read at 595 nm. For the blank, only 800 μL of distilled water and Bradford reagent were used.

### 2.7. Total Lipid Extraction and Methyl Esterification

Whole brain tissue was homogenized in a cold mortar, with 0.1 g of this homogenate taken and mixed with 2 mL of a 2:1 (*v*/*v*) chloroform–methanol solution in a cold mortar. The homogenate was collected and centrifuged at 6000 rpm for 10 min at 4 °C. The supernatant was collected, and 100 µL of water was added. The mixture was centrifuged at 3000 rpm for 20 min at 4 °C, and the lower phase was recovered and stored at −20 °C. Methyl esterification was performed using 100 µL of the lower phase and 1 mL of 0.5 N NaOH in methanol. It was incubated at 100 °C for 60 min. Then, 1 mL of boron trifluoride (BF_3_) dissolved in methanol was added and incubated for an additional 20 min at 100 °C. After cooling, the contents were transferred to a test tube, and 2 mL of water and 3 mL of hexane were added. The mixture was mixed for 20 s, and the upper (organic) phase was recovered and placed in a second test tube, where an additional 3 mL of hexane was added. The mixture continued for another 20 s, and the organic phase was collected once more. Finally, the organic phase was dried using a stream of nitrogen and resuspended in isooctane. The samples were analyzed using an Agilent 7890A gas chromatography system (Agilent Technologies, Santa Clara, CA, USA) equipped with an HP5MS30M column (5% phenyl methylsiloxane, 60 × 0.25 × 0.25 mm; Agilent Technologies, Santa Clara, CA, USA) and coupled to an electronic impact ionization quadrupole mass spectrometer. Hewlett-Packard 5975C (Santa Clara, CA, USA). The oven temperature was initially set at 60 °C for 1 min, then increased to 280 °C at a rate of 8 °C/min. The injector, ionization source, and quadrupole temperature were maintained at 230 °C, 230 °C, and 150 °C, respectively. Helium served as the carrier gas at a constant flow of 1 mL/min. The mass spectrometer operated in EI mode at 70 eV, with a mass-to-charge (m/z) range of 50–500, and the voltage was set to −1737 V. Total ion chromatograms (TICs) were processed using the automated data processing software MSChem (Version E.02.01.1177, Agilent Technologies). To identify the different compounds, the mass spectrum of each detected compound was compared to those in mass spectral databases (Wiley 275 and US National Institute of Science and Technology (NIST) V. 2.0). The quantities of compounds were calculated from a standard calibration curve using 1,8-cineole in the range of 1–0.2 mg/mL.

### 2.8. Parallel Artificial Membrane Permeability Assay Blood–Brain Barrier (PAMPA BBB)

The PAMPA BBB assay kit was used as in vitro model to predict transcellular passive absorption. The assay is cost-effective, quick, and mimics the three barriers: skin, gastrointestinal, and blood–brain). The concentration and dose were selected based on the physicochemical properties of the active pharmaceutical ingredients, rather than on relevant in vivo dose data. The donor volume-to-membrane area ratio is also significantly higher compared to the in vivo environment. Permeability is determined and used for assay evaluation, which is normalized to the donor concentration [29].

Approximately 3 µL of PBL (porcine brain lipids) at a concentration of 5 mg/mL dissolved in dodecane was applied to the surface of the artificial membrane in the donor plate. Subsequently, 297 µL of 100 mM phosphate-buffered saline was added to the donor plate, along with 300 µL to the acceptor plate. Then, 3 µL of the compound, in this case, the *C. citrinus* extract at a concentration of 100 mg/mL dissolved in 5% DMSO, was added to the donor plate. The donor plate was carefully placed on the acceptor plate and incubated for 14 to 18 h at 37 °C. After the incubation period, the contents of the acceptor plate were collected in a microtube (Eppendorf, Hamburg, Germany). Then, 300 µL of chloroform was added to this content and vortexed for 5 min to extract the compounds in the buffer. The organic phase was recovered and placed in vials for analysis using gas chromatography coupled with mass spectrometry. The 1,8-cineole (Sigma-Aldrich) was used as an internal standard. The same protocol was followed for the intestinal barrier permeability test, with a slight modification in the membrane compounds, using soy lecithin (20 mg/mL) dissolved in chloroform.

### 2.9. Advanced Oxidation Protein Products (AOPP)

The reaction mixture consisted of 50 µL of tissue homogenate and 1 mL of 20 mM phosphate buffer at pH 7.4, then 50 µL of 1.16 M potassium iodide and 100 µL of acetic acid were added. After a 2 min incubation, the absorbance at 340 nm was measured [30].

### 2.10. Malondialdehyde (MDA) and 4-Hydroxynonenal (4-HNE)

The assay for malondialdehyde (MDA) and 4-hydroxynonenal (HNE) was based on using 1-methyl-2-phenylindole, as reported by Johnston et al. [31]. A mixture containing 0.5 g of tissue, 10 μL of 5 mM BHT, and 2 mL of 10 mM phosphate buffer at pH 7.4 was homogenized in a cold mortar with a pestle. The homogenate was centrifuged for 13 min at 13,000× *g*, and the supernatant was collected. For the assay, 200 μL of tissue homogenate was mixed with 650 μL of 10 mM 1-methyl-2-phenylindole dissolved in a 3:1 acetonitrile/methanol solution, and the sample was prepared in duplicate. One sample was treated with 150 μL of 37% HCl (*v*/*v*), while the other was treated with 150 μL of methanesulfonic acid. Both samples were incubated for 60 min at 45 °C. After incubation, the samples were placed on ice for 5 min, then centrifuged for 10 min at 13,000× *g*. The supernatant was read at 586 nm. For the blank, the 1-methyl-2-phenylindole solution was replaced with a 3:1 acetonitrile/methanol mixture (one blank for each acid).

### 2.11. Reduced Glutathione (GSH)

Reduced glutathione (GSH) levels in brain tissue were measured using the method described by Sedlak and Lindsay [32]. A mixture of 62.5 μL of tissue homogenate, 187.5 μL of 0.2 M Tris buffer at pH 8.2, and 12.5 μL of 0.01 M 5,5′-dithiobis(2-nitrobenzoic acid) (DNTB) was prepared, followed by the addition of 987.5 μL of absolute methanol. The mixture was then shaken at 240 rpm for 15 min. Afterwards, the samples were centrifuged at 3000 rpm at room temperature for 15 min. Finally, the yellow color of the reaction developed, and the absorbance was measured with a spectrophotometer at 412 nm.

### 2.12. Catalase (CAT) Activity

Catalase (CAT) activity was assessed by measuring the rate of hydrogen peroxide (H_2_O_2_) decomposition [33]. The reaction mixture contained 950 µL of 50 mM phosphate buffer at pH 7.0, 25 µL of brain homogenate, and 25 µL of 30 mM H_2_O_2_ substrate. Changes in absorbance at 240 nm were recorded every 30 s over 3 min. The blank consisted of 975 µL buffer and 25 µL H_2_O_2_. The molar extinction coefficient for peroxide was 43.6 M^−1^cm^−1^.

### 2.13. Glutathione Peroxidase (GPx) Activity

Glutathione peroxidase activity was measured following the protocol of Prabhu et al. [34]. A working solution included 10 µL of 0.5 mM NADPH, 10 µL of 100 mM reduced glutathione, 1 unit of glutathione reductase enzyme, 901 µL of 50 mM phosphate buffer at pH 7.0, 50 µL of 30 mM cumene hydroperoxide, and 25 µL of brain homogenate. The absorbance was measured at 340 nm every 30 s over 5 min against a blank containing all components except the sample and glutathione reductase.

### 2.14. Superoxide Dismutase (SOD) Activity

SOD activity was measured using the technique described by Bouhalit et al. [35], conducted in darkness or with minimal light exposure. For this method, the following were added to a microtube (Eppendorf): 542.5 μL of 67 mM phosphate buffer (pH 7.8), 25 μL of 100 mM EDTA, 50 μL of 50 mM sodium carbonate (Na_2_CO_3_) at pH 10, 7.5 μL of TEMED, 200 μL of 1.5 mM NTB, 125 μL of 20 mM methionine dissolved in 2% DMSO, 50 μL of brain homogenate, and finally 25 μL of 0.1 mM riboflavin. Two microtubes (Eppendorf) served as blanks; all the components were added except for the sample. All tubes were illuminated, except for one unilluminated blank tube, with a 100 W spotlight placed 15 cm away for 15 min. The measurement was then taken at 560 nm in a spectrophotometer.

### 2.15. Paraoxanase (PON) Activity

Assessing PON was performed following Dantoine’s protocol [36]. A reaction mixture was prepared with 25 µL of methanol, 100 µL of 10 M CaCl_2_, 562.5 µL of 25 mM Tris-HCl buffer at pH 8, and 312.5 µL of 1 M 4-nitrophenylacetate dissolved in methanol. A volume of 125 µL (1/20 dilution) of brain homogenate was added to a glass cell, followed by 1 mL of the prepared cocktail. The cell was shaken and read in a spectrophotometer at 402 nm every 30 s for 3 min against a blank of the cocktail.

### 2.16. Statistical Analysis

The entire sample (*n* = 6) was included in the treatment, and values were expressed as mean ± standard deviation (SD). Data were analyzed using JMP 8.0 and GraphPad Prism (version 7) with one-way analysis of variance (ANOVA) to identify statistical differences in parameters. Tukey’s multiple comparison test (a, b, c) was performed. Tukey’s Honestly Significant Difference (HSD) test is a post hoc test used in ANOVA to compare all possible pairs of means. When ANOVA reveals a significant difference among group means, a post hoc test, such as Tukey’s, is necessary to determine which groups differ significantly. Values of * *p* ≤ 0.05 were considered statistically significant.

## 3. Results

**Figure 1:**
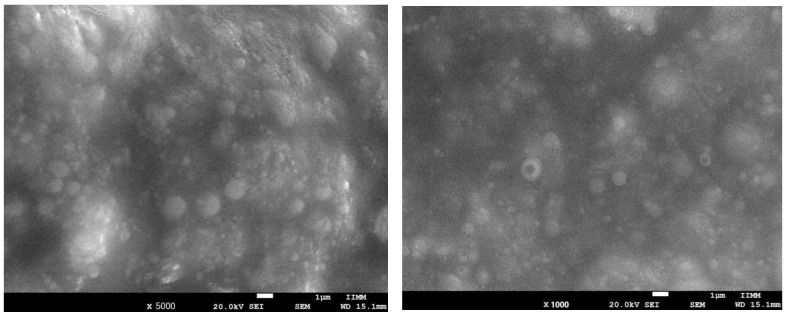
Scanning electron microscope images at 5000× (**left**) and 1000× (**right**) of phytosomes loaded with *Callistemon citrinus* leaf extract.

The use of scanning electron microscopy (SEM) has given significant insight into the solid-state properties and surface morphology of complex (Figure 1). Particles are small and adjoining; this size range can be used for oral absorption.

The size of the phytosomal formulation of *C. citrinus* prepared in this study was 129.98 nm ± 18.30 nm in the emulsion. The entrapment efficiency was 80.49 ± 0.07. The stability of the phytosomal formulation lasted 3.5 months at 20 °C and showed broad solubility (Table 2).

### 3.1. Effects of C. citrinus Phytosomes on Overall Weight Gain

**Figure 2:**
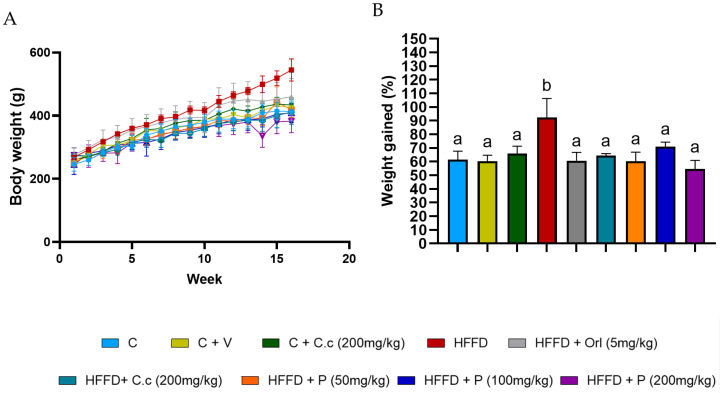
Weekly body weight in control rats and the different experimental treatments during the 16 weeks (**A**), and the percentage of total weight gained by control rats and various experimental treatments over 16 weeks is shown (**B**). Values are expressed as mean ± standard deviation (ANOVA followed by Tukey’s test, *n* = 6). Different letters (a,b) indicate significant differences between groups.

Figure 2A,B shows that weight gain in the HFD group started to increase in the 4th week, and by the end of the 16th week, it was 267.33 ± 36.67 g, while the control group’s gain was 171.0 ±36.4 g (*p* < 0.0014). This represents a 30% increase in body weight compared to the group that received the same high-fat–fructose diet supplemented with *C. citrinus* extract, phytosomes, and orlistat. The lower doses of phytosomes (50 mg/kg) displayed a pattern similar to that of the *C. citrinus* extract at 200 mg/kg. Table 3 presents some morphometric parameters that demonstrate the impact of a high-fat, high-fructose diet. The Adiposity index was higher in the HFD group, 9.43 ± 0.623, than the control group, 2.78 ± 0.55 (*p* < 0.03), while in the HFD supplement with *C. citrinus* at 200 mg/kg, phytosomes + *C. citrinus* at 200 mg/kg, and orlistat at 5 mg/kg, the AI was 6.19 ± 0.39, 4.02 ± 0.62, and 5.56 ± 0.7 (*p* rate 0.001 to 0.021), respectively. On the other hand, the only group with a Lee index of 0.33 (*p* < 0.05) was the HFD group; this value indicates obesity in the rodents, unlike the other groups. This finding suggests that a potential mechanism for managing weight gain in *C. citrinus* and the phytosomes is comparable to that of orlistat.

### 3.2. Brain Lipid Profile

**Figure 3:**
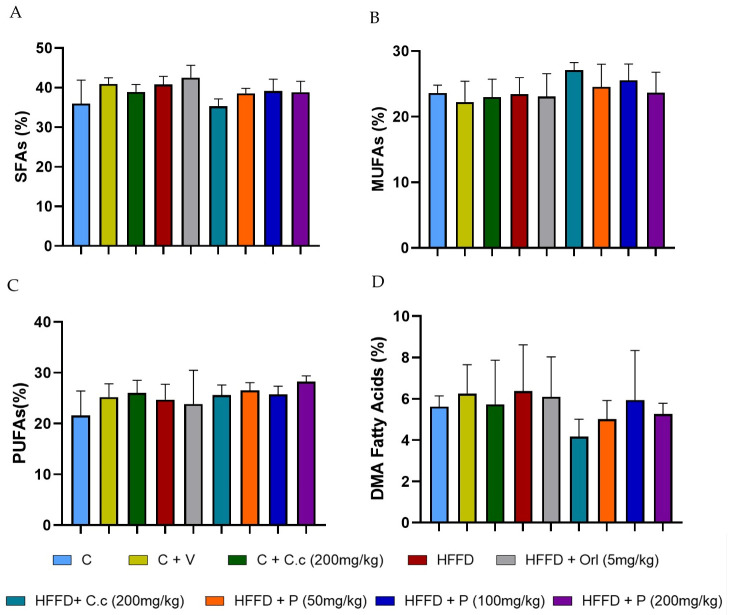
Effect of *Callistemon citrinus* extract and phytosomes on the lipid profile in brain tissue, (**A**) saturated fatty acids (SFAs), (**B**) monounsaturated fatty acids (MUFAs), (**C**) polyunsaturated fatty acids (PUFAs), and (**D**) dimethyl acetaldehyde (DMA) fatty acids. Control (C); control + vehicle (C + V); control + *C. citrinus* leaf extract (C + C.c, 200 mg/kg), high-fat diet (HFD), HFD + orlistat (HFD + Orl, 5 mg/kg), HFD + *C. citrinus* leaf extract (HFD + C.c, 250 mg/kg), HFD + phytosomes (HFD + P, 50 mg/kg, 100 mg/kg, and 200 mg/kg, respectively). Values are expressed as mean ± standard deviation (ANOVA followed by Tukey’s test, *p* < 0.05, *n* = 6).

To evaluate the effect of *Callistemon citrinus* extract and the phytosome on the total brain lipid composition, we analyzed the whole brain. The lipid profile results show no significant differences in the percentages (*p* < 0.05) of saturated fatty acids (Figure 3A), monounsaturated fatty acids (Figure 3B), polyunsaturated fatty acids (Figure 3C), and dimethyl acetal fatty acids (Figure 3D) when comparing the control and HFD groups to other treatments after 16 weeks. Additionally, the main fatty acids found in brain tissue across various treatments include palmitic acid (16:0), stearic acid (18:0), arachidonic acid (ARA) (20:4), and 4,7,10,13,16,19-docosahexaenoic acid (DHA) (22:6) (Table 4). A high level of palmitic acid was observed in the HFD + Orl group, but it was not significantly different (*p* < 0.05) from the control group. However, the other groups did not differ substantially from one another. Stearic acid levels showed a significant (*p* < 0.05) increase in the HFD + Orl (5 mg/kg). Conversely, in the HFD + C.c (200 mg/kg), there was a significant (*p* < 0.05) reduction compared to the other groups. Palmitoleic acid levels in the HFD group increased significantly (*p* < 0.05) compared to the different groups. The high-fat–fructose diet did not produce significant changes in the percentages of ARA (20:4) and DHA (22:6) across all the treatments. On the other hand, no effects were observed in the profiles of DMA compounds.

### 3.3. Parallel Artificial Membrane Permeability Assay (PAMPA)

**Figure 4:**
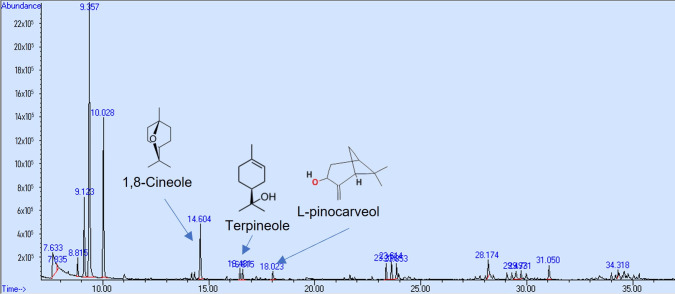
GC/MS chromatogram (arbitrary units) of *C. citrinus* leaf extract showing compounds that could cross the intestinal barrier in vitro.

**Figure 5:**
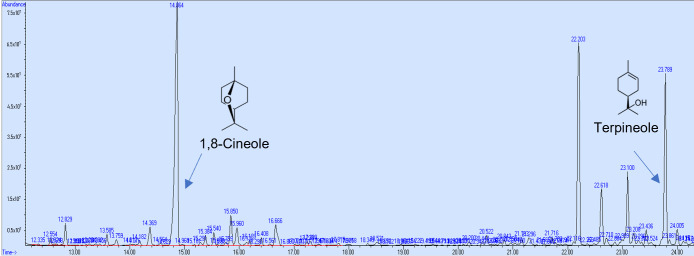
GC/MS chromatogram (arbitrary units) of *C. citrinus* leaf extract showing compounds that could cross the blood–brain barrier in vitro.

In the research to use natural products with neuroprotective effects, the first step is to demonstrate their permeability through the BBB using the PAMPA, and thus select the compounds that can access the brain. However, a pharmacokinetics study in vivo is required to demonstrate the compound’s bioavailability. In this study, the PAMPA was used to identify components of *Callistemon citrinus* capable of crossing the intestinal and blood–brain barriers (BBBs). 1,8-Cineole exhibited partial permeability in the artificial intestinal barrier assay, with a concentration of 0.024 mg/mL. Meanwhile, α-terpineol and L-pinocarveol were detected at very low levels (Figure 4). Conversely, in the BBB permeability assay, 1,8-cineole and α-terpineol demonstrated higher permeability, with concentrations of 0.094 mg/mL and 0.016 mg/mL, respectively (Figure 5).

### 3.4. Biomarkers of Oxidative Stress

**Figure 6:**
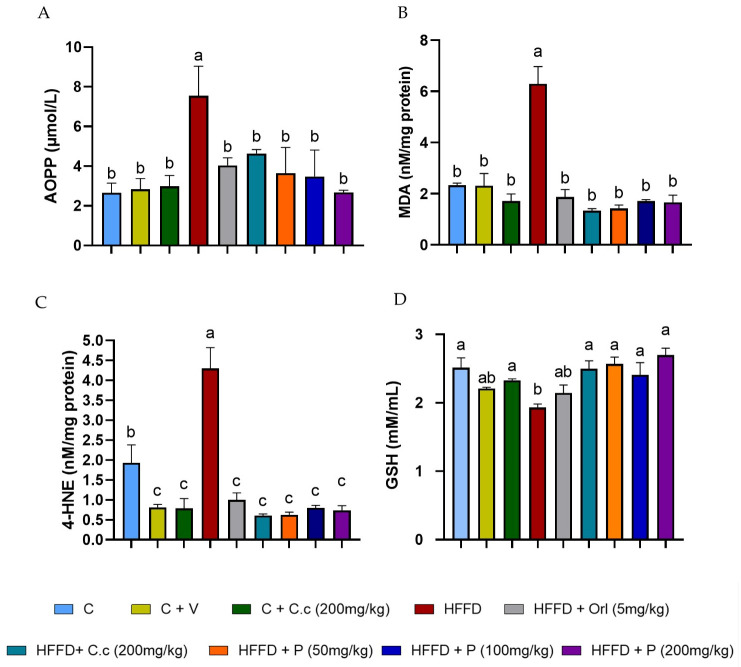
Effect of *Callistemon citrinus* extract and phytosomes on the generation of oxidative stress markers. Advanced oxidation protein products (AOPPs) (**A**), malondialdehyde (MDA) (**B**), 4-hydroxynonenal (4-HNE) (**C**), and reduced glutathione (GSH) (**D**). Control (C), control + vehicle (C + V), control + *C. citrinus* leaf extract (C + C.c, 200 mg/kg), high-fat diet (HFD), HFD + orlistat (HFD + Orl, 5 mg/kg), HFD + *C. citrinus* leaf extract (HFD + C.c, 250 mg/kg), HFD + phytosomes (HFD + P, 50, 100, and 200 mg/kg, respectively). Values are expressed as mean ± standard deviation (ANOVA followed by Tukey’s test, *p* < 0.001, *n* = 6). Different letters (a, b, c) indicate statistically significant differences between groups.

Figure 6A–C shows that the high-fat diet (HFD) group showed high levels of advanced oxidation protein products (AOPPs) and lipid peroxidation products, such as 4-hydroxynonenal (4-HNE) and malondialdehyde (MDA), as compared to the other groups (*p* < 0.0001). However, the level of 4-HNE in the control group was higher than in the treatment groups (*p* < 0.0001), which showed significantly lower levels of 4-HNE.

Finally, the HFD group exhibited significantly lower levels of reduced glutathione (GSH) compared to the control, *C. citrinus*, HFD-treated *C. citrinus*, and phytosomes (*p* values ranging from 0.0001 to 0.016). Meanwhile, the groups supplemented with vehicle and HFD + Orlistat showed no statistically significant differences compared to the HFD-alone groups (Figure 6D). The groups that received a high-fat diet supplemented with the extract and phytosomes from *C. citrinus* exhibited a decrease in reactive oxygen species (ROS) production, thereby preventing oxidative stress in the brain.

### 3.5. Antioxidant Enzyme Activities

**Figure 7:**
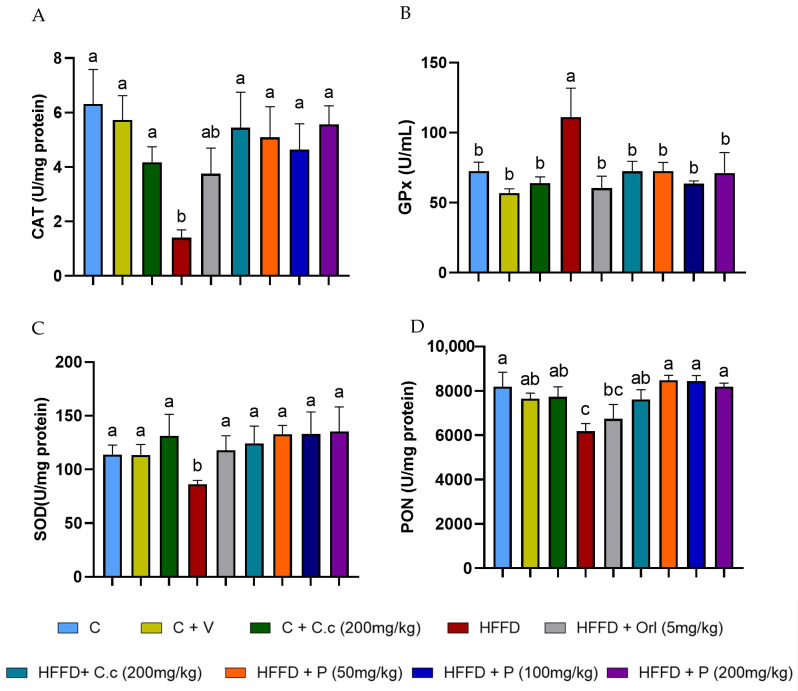
Effect of *C. citrinus* leaf extract and phytosomes on the activity of catalase (CAT) (**A**), glutathione peroxidase (GPx) (**B**), superoxide dismutase (SOD) (**C**), and paraoxonase (PON) (**D**) in brain tissue. Control (C), control + vehicle (C + V), control + *C. citrinus* leaf extract (C + C.c, 200 mg/kg), high-fat diet (HFD), HFD + orlistat (HFD + Orl, 5 mg/kg), HFD + *C. citrinus* leaf extract (HFD + C.c, 250 mg/kg), HFD + phytosomes (HFD + P, 50, 100, and 200 mg/kg, respectively). Values are expressed as mean ± standard deviation (ANOVA followed by Tukey’s test, *p* < 0.001, *n* = 6). Different letters (a, b, c) indicate statistically significant differences between groups.

CAT activity in the HFD group showed a statistically significant 77% decrease (*p* < 0.05) compared to the control group (*p* < 0.0001). On the other hand, the HFD group supplemented with orlistat also showed a 55% reduction in CAT activity compared to the control group (*p* < 0.05). However, the groups supplemented with *Callistemon citrinus* extract and phytosones at the three different doses reduced the impact of the high-fat–fructose diet, maintaining CAT activity levels similar to the control group (Figure 7A). Additionally, as shown in Figure 7C, SOD activity in the brain of the HFD group was significantly reduced by 24% compared to both groups without HFD and those fed HFD but supplemented with *C. citrinus*, phytosome, and orlistat (*p* values ranging from 0.0003 to 0.007). Conversely, the PON activity of the HFD group revealed a statistically significant 20% decrease compared to the control and HFD groups supplemented with phytosomes at three doses (*p* values ranging from 0.0004 to 0.0017). In contrast, the groups with vehicle, *C. citrinus* extract without and with diet were slightly lower but not statistically significant compared to the control group. Analysis of the HFD group treated with orlistat again showed no significant difference between the HFD group and the other groups (Figure 7D).

Unlike other enzymes, the levels of GPx activity exhibit a significant 2-fold increase in the HFD group compared to all other groups ( *p* values ranging from 0.0001 to 0.027) (Figure 7B). Our results showed that, regardless of the phytosome concentration, all treatments had the same effect on the oxidative stress marker and antioxidant enzymes. However, phytosomes loaded with 50 mg/kg of *C. citrinus* extract demonstrated similar antioxidant potential to higher doses, suggesting that a lower dose might be more effective for achieving therapeutic benefits.

## 4. Discussion

This study utilized scanning electron microscopy (SEM), which provided valuable insights into the solid-state properties and surface morphology of complexes. In future studies, transmission electron microscopy (TEM) could be used to reveal surface topography along with detailed internal structure and crystallographic information sample. SEM analyses do not allow for definitive conclusions about the formation of phytosomes. Therefore, it is better to describe it as a phospholipid complex formulation with potential phytosomes characteristics. However, the phospholipid complex formulation prepared in this study was able to encapsulate 80.49 ± 0.07% of C. citrinus extract and had a size range of 129.98 ± 18.30 nm, which indicates that this small particle size is suitable for oral absorption. Additionally, the use of soybean phosphatidylcholine, which has a higher absorption rate, enables this formulation to be used orally administration.

Research findings confirm that *Callistemon citrinus* extract and its phytosomes formulation can help prevent weight gain and reduce oxidative stress in the brains of rats consuming a high-fat, high-fructose diet for 16 weeks. Previously, we reported that the anti-obesogenic, antioxidant, and anti-inflammatory properties of *C. citrinus* are due to its major bioactive compounds [20]. Ayala-Ruiz et al. [24] demonstrated that supplementation with 1,8-cineole, α-terpineol, limonene, and a combination of these terpenes decreases body weight gain in rats fed a high-fat–sucrose diet, and their behavior closely resembles that of the control group. Moreover, previous studies have suggested that the potential anti-obesogenic mechanism of *C. citrinus* may be linked to the inhibition of pancreatic lipase [20].

The increase in the intake of saturated and trans fats, along with excess sucrose or fructose, contributes to overweight or obesity and associated conditions, including type 2 diabetes, hyperlipidemia, hypertension, and hypercholesterolemia [37]. The accumulation of adipose tissue results in increased levels of free fatty acids in the blood and other tissues. High-fat, high-carbohydrate diets have been shown to alter the lipid profiles of organs such as the liver, heart, and skeletal muscle, resulting in higher levels of saturated fatty acids (SFAs) and a decrease in polyunsaturated fatty acids (PUFAs) [38,39,40]. The brain is the second tissue with a high content of lipids, but it has a lower antioxidant system; therefore, a rise in fatty acid levels from the diet results in an increase in ROS due to β-oxidation [41].

Kaplan and Greenwood [5] first reported the relationship between high-fat intake and cognitive impairment in rats fed soybean oil or lard. Conversely, Da Silva-Santi et al. [42] demonstrated that brain fatty acids are affected in mice fed high-fat or high-carbohydrate diets for 7, 14, 28, or 56 days. They observed that, initially, differences in fatty acid composition were evident; however, by the end, these differences had leveled out. Inflammatory markers were higher in the high-fat diet group compared to the high-carbohydrate diet, while markers of microglial infiltration were more prominent in the high-carbohydrate diet group. These diets have been linked to changes in the brain and are associated with the promotion of neuroinflammation [43]. Sighinolfi et al. [44] demonstrated that diets containing 60% fat (40% SFA) induce lasting changes in the lipid composition of specific brain regions, including the cortex, striatum, and hypothalamus—areas crucial for eating behavior and responses related to peripheral metabolism.

Although lipid composition is tightly regulated in the brain, this study aimed to determine whether phytosomes loaded with *Callistemon citrinus* affect the fatty acid profile in the brains of rats fed a diet rich in animal and vegetable fats, along with added fructose for 16 weeks. The primary fatty acids in the brain are arachidonic acid (ARA, 20:4*n*-6) and docosahexaenoic acid (DHA, 22:6*n*-3). Since the brain’s de novo synthesis of polyunsaturated fatty acids (PUFAs) is limited, these fatty acids must be obtained from the diet or their precursors, α-linolenic acid (ALA, 18:3*n*-3) and linoleic acid (LA, 18:2*n*-6), which are metabolized into PUFAs in the liver. Conversely, eicosapentaenoic acid (EPA, 20:5*n*-3), ALA, and LA are generally found at lower concentrations in the brain compared to arachidonic acid (ARA) and docosahexaenoic acid (DHA).

Despite differences in treatment, this study found that the overall brain fatty acid profile across all groups showed minor changes in fatty acid concentrations. This aligns with the findings reported by Reichlmayr-Lais et al. [45], who examined the effects of various vegetable oil fats. The percentages of fatty acids (C16:0, C18:0, C20:4, and C22:6) observed in our study were similar to those previously reported in the brain [46]. The group that received a high-fat–fructose diet supplemented with *C. citrinus* extract showed values identical to those of the control group. In contrast, the other groups had slightly higher values than these two groups (Table 3, Figure 4). Additionally, the fatty aldehydes in the brain, such as C16:0 dimethyl acetaldehyde (DMA), C18:0 DMA, and C18:1 (*n*-7 or *n*-9), showed no significant differences between groups.

Although several studies report small changes in the brain lipid profile as a function of brain region, this study quantified the profile for the entire brain due to the limitation established by the University’s Bioethical Committee Guidelines regarding the number of animals that can be used in these studies. Unfortunately, given the number of Wistar rats used in this study, it was not possible to determine the fatty acid profile by brain region.

Researchers have identified numerous natural products with antioxidant and anti-inflammatory properties; however, few can cross the blood–brain barrier when present in complex mixtures. This study used the in vitro parallel artificial membrane permeability assay to identify potential compounds in *Callistemon citrinus* that might cross the intestinal and blood–brain barriers. Mony et al. [47] described the ability of terpenoid compounds to cross the blood–brain barrier (BBB), attributing their restorative effects to damage caused by oxidative stress within it, thereby normalizing its function and promoting brain health. Jäger et al. [48] revealed that 1,8-cineole can enter the body through inhalation, with its highest concentration in blood serum occurring at 18 min after exposure. They also demonstrated that body fat influences the half-life of cineole, as it is eliminated more quickly by organisms with lower body fat. Our findings suggest that 1,8-cineole and α-terpineol can permeate both the intestinal and blood–brain barriers in vitro assays. This monoterpene has also shown effectiveness against inflammatory intestinal diseases, such as Crohn’s disease and ulcerative colitis, by increasing the activity of the PPARγ receptor [49]. According to Satou et al. [50], other monoterpenes such as limonene, α-pinene, and linalool can also be found in brain tissue after inhalation. However, in this study, we were unable to detect limonene as a compound capable of permeating the brain through the PAMPA BBB assay, despite its presence in the *C. citrinus* extract. Dao et al. [51] confirmed that 1,8-cineole can cross the blood–brain barrier (BBB), even when administered orally. These findings suggest that terpenes from *C. citrinus* may help enhance the brain’s antioxidative status. However, a future pharmacokinetic study of compounds in the plasma or brain following oral administration will be considered.

*Callistemon citrinus* derives its antioxidant properties mainly from various secondary metabolites, including 1,8-cineole, limonene, and α-terpineol [22], as well as phenolic compounds and flavonoids [23]. Ayala-Ruiz et al. [24] demonstrated that 1,8-cineole, limonene, and α-terpineol, both alone and in combination, exhibit strong antioxidant effects by reducing oxidative stress markers (MDA, HNE, AOPP) and pro-inflammatory cytokines (TNFα, IL-6, and leptin) in rat livers fed a high-fat–sucrose diet. In our study, we observed that oxidative stress in brain tissue was triggered in the group fed a high-fat–fructose diet, leading to an increase in lipid peroxidation products, specifically MDA and 4-HNE. Meanwhile, MDA is believed to be mutagenic, and 4-HNE is especially toxic due to its reactivity with thiol and amino acid groups [52]. As reported by Hou et al. [53], the concentration of MDA rises in the brains of rats consuming a high-fat diet. Similarly, Maciejczyk et al. [54] found significantly higher levels of MDA and 4-HNE in the cortex and hypothalamus of rats on a high-fat diet compared to the control group.

Another biomarker commonly used to measure oxidative stress is advanced protein oxidation products (AOPPs). These mainly consist of damaged protein aggregates, often containing dityrosine residues that contribute to crosslinking, disulfide bonds, and carbonyl groups. Initially, AOPP were only detected in plasma, formed by the reaction of plasma proteins (albumin) with chlorinated oxidants, which are products of myeloperoxidase activity [30]. However, AOPP levels are also reported in the cerebrospinal fluid, liver, kidney, and brain [55,56,57,58]. Furthermore, elevated levels of AOPP have been found in the brains of people with Alzheimer’s disease [59]. In this study, a significant increase in AOPP levels was observed in the HFD group compared to the other groups. The groups fed an HFD and supplemented with the extract and phytosomes of *Callistemon citrinus* showed a reduction in oxidative stress biomarkers (MDA, 4-HNE, and AOPP), making them statistically comparable to the control group.

Reduced glutathione (GSH) is a tripeptide vital for the brain, playing a key role in detoxifying reactive oxygen species (ROS). The brain requires a substantial amount of oxygen, making it particularly susceptible to the formation of reactive species [60]. Additionally, it is the second most tissue rich in polyunsaturated fatty acids. Moreover, the antioxidant system in the brain is weaker than in other tissues, increasing its vulnerability to oxidative stress [61]. Alzoubi et al. [62] noted a decline in GSH levels in the cortex but found no differences in the hippocampus and cerebellum of rats fed a high-fat diet. Similarly, Cavaliere et al. [63] reported a significant decrease in reduced glutathione levels in the brains of mice on a high-fat diet. These findings support our results and highlight the effectiveness of *C. citrinus* extract and phytosomal formulation in reducing oxidative stress caused by a high-fat diet (HFD) in the brains of Wistar rats.

The primary enzymatic defense against oxidative stress involves three enzymes: SOD, which catalyzes the dismutation of superoxide anion radicals into hydrogen peroxide, followed by CAT and GPx, the enzymes that reduce hydrogen peroxide, thereby preventing the accumulation of harmful reactive species ROS. However, these enzymes are affected by HDF feeding in different ways. Liu et al. [64] reported a decrease in the expression of SOD and CAT in the brain of mice fed a high-fat diet for 6 months. Similarly, El Azab and Abdulmalek [65] showed significant reductions in the levels of both enzymes in the brains of both young and aged rats on a high-fat diet. Our study shows that oral supplementation with the extract and phytosomes of *C. citrinus* increases SOD and CAT activities in the brain, even in rats fed a high-fat diet (HFD), which contrasts with the findings in the HFD group. This result highlights that *C. citrinus*, whether extracted or in phytosome form, exhibits a significant antioxidant effect in the brain.

The third enzyme, GPx, catalyzes the conversion of peroxides and hydroperoxides into water or alcohols. GPx plays a crucial role in maintaining cellular redox balance and protecting neural cells from oxidative stress [66]. The activity of this enzyme decreases in obesity models; for example, Amri et al. [67] reported low GPx activity levels in the brains of Wistar rats fed a high-fat, high-fructose diet for 12 weeks. Similarly, Charradi et al. [68] observed reduced glutathione peroxidase (GPx) enzyme activity in the brains of rats fed a high-fat diet for 6 weeks. Conversely, Savaskan et al. [69] demonstrated an overexpression of GPx4 (cytosolic) in response to brain damage. An increase in GPx activity has been linked to higher levels of lipid peroxidation products [70]. In our study, elevated levels of MDA and 4-HEN were found only in the high-fat–fructose diet (HFD) group, which was associated with an increase in GPx. Conversely, all groups consuming an HFD supplemented with extracts and phytosomes of *C. citrinus* exhibited GPx activity similar to that of the control group. A previous study by Ortega-Pérez et al. [21] found that HFD increases GPx activity in the liver and heart, which is associated with decreased GSH levels and elevated levels of MDA, 4-HNE, and AOPP.

Paraoxonase is another enzyme used as a biomarker of oxidative stress; anti-inflammatory activity has been reported in models of Alzheimer’s disease and hyperlipidemia, where reduced PON activity has been observed [71]. Shih et al. [72] reported that PON2 is essential for preventing obesity. The first reported PON activity involves protecting LDL and HDL against lipid peroxidation. Nguyen and Sok [73] reported that monoenoic acid inhibited its activity. In our study, the HFD group exhibited a high concentration of palmitoleic acid as well as elevated levels of 4-HNE, which is also known to inhibit PON activity. Meanwhile, supplementation with *C. citrinus* extract and phytosomes helps reduce the decline of palmitoleic acid and 4-HNE levels, maintaining PON activity similar to that of the control group.

Orlistat is a medication used to assist weight loss mainly by blocking pancreatic lipase, which prevents fats from being absorbed into the intestine. Zakaria et al. [74] demonstrated that orlistat can help prevent insulin resistance and oxidative stress, and it protects the liver from damage caused by increased fat intake during a high-fat diet. Othman et al. [75] reported that orlistat can reduce oxidative stress in the myocardial tissue of obese rats. Mahmoudi et al. [76] demonstrated that orlistat, when combined with grape seed extract, resulted in a reduction in brain lipoperoxidation. AL-Dalaeen et al. [77] showed that orlistat has beneficial anti-inflammatory effects on the brain.

In our study, orlistat was administered via oral gavage simultaneously with the induction of obesity, whereas in other studies, obesity was first established before treatment. The HFD plus orlistat group showed a reduction in weight gain but experienced loose stools. In contrast, the group fed HFD supplemented with *C. citrinus* extract and phytosomes reduced body weight without this side effect. The antioxidant activity of orlistat in this study was found to be similar, based on levels of MDA, 4-HNE, and AOPP, with an increase in SOD and GPx activity compared to the treatment groups with *C. citrinus*. However, orlistat had minimal effects on CAT, PON, and GSH. Our results support the evidence that a high-fat–fructose diet is closely linked to brain oxidative stress, characterized by increased levels of MDA, 4-HNE, and AOPP, as well as decreased levels of GSH. Meanwhile, the extracts and phytosomes of *C. citrinus* exhibited antioxidant properties, which lowered the biomarkers of oxidative stress (MDA, 4-HNE, and AOPP) and increased the enzymatic antioxidant activity of CAT, SOD, and PON1, while also raising the levels of GSH.

Most of the observed antioxidant activity is attributed to the major terpenes and phenols in *C. citrinus*, including 1,8-cineole, α-terpineol, gallic acid, p-coumaric acid, and ellagic acid [18,21]. 1,8-Cineole and α-terpineol have been demonstrated to exhibit antioxidant and neuroprotective effects in the brain [78,79]. Gallic acid provided a protective effect on the blood–brain barrier (BBB) [80]. Conversely, p-coumaric and ellagic acids demonstrated both antioxidant and anti-neuroinflammatory impact in the brain [81,82].

Although phytosomes improve the bioavailability, solubility, and stability of compounds in the extract, no significant differences were observed among the various phytosomes loaded with C. citrinus at doses 50, 100, and 200 mg/kg, or when compared to the extract at 200 mg/kg, in their ability to reduce oxidative stress in the brain. One possible explanation is that, according to the in vitro PAMPA, 1,8-cineole and α-terpineol are able to cross both the intestinal and blood–brain barriers. These compounds may help prevent oxidative stress in the brains of Wistar rats fed a high-fat–fructose diet. However, using a lower dose of phytosomes to achieve the same effect as a higher dose is a promising approach. Using male Wistar rats simplified the process because, in female rats, hormone fluctuations significantly affect some parameters. However, future studies should consider female rats in the tests. Another limitation of the study was the small number of animals, which prevented the examination of the fatty acid profile in different brain regions and the extension of the study period to collect samples at specific times. Future studies should consider dose-dependent tests, evaluate animal behavior, assess pro-inflammatory cytokines, such as TNF-α, IL-6, and IL-1β, along with leptin levels, as well as enzymes involved in the inflammatory process, including myeloperoxidase, cyclooxygenase-2, 5-lipoxygenase, and xanthine oxidase. Finally, analyzing the expression of antioxidative enzymes and testing the major compounds should be interesting to validate the possible mechanism of action of *Callistemon citrinus* extract and phytosomes act.

## 5. Conclusions

Our findings provide the first evidence that a high-fat–fructose diet causes oxidative stress in the brain, which can be prevented by administering phytosomes loaded with *Callistemon citrinus* extracts at a lower concentration than the pure extract. Moreover, our study shows that 1,8-cineole and α-terpineol, the main bioactive compounds identified in *C. citrinus*, are likely responsible for its antioxidant effects. Therefore, the phytosomes loaded with *C. citrinus* might be seen as a promising supplement to prevent oxidative stress in the brain. In future studies, it would be advisable to perform toxicology and pharmacokinetic studies of phytosomes to gather additional data that supports the use of this formulation in combating oxidative stress in the brain caused by obesity.

## Figures and Tables

**Table 1:** Percentage of fatty acid composition in the fat diet.

Fatty Acid	Lard (%)	Vegetable Shortening (%)
C10:0, Capric	-	0.16
C12:0, Lauric	0.26	0.27
C13:0, Tridecanoic	-	0.067
C13:1, Tridecenoic	-	0.13
C14:0, Myristic	2.6	5.4
C14:1*n*-6	-	0.3
C14:2*n*-9	-	0.72
Tetradecanoic acid, 12-methyl	-	0.57
Pentadecanoic acid	0.14	1.01
C16:0, Palmitic	22.8	16.4
C16:1, Palmitoleic	5	2.27
Methyl 14-methylhexadecanoate	-	0.47
C17:0, Margaric	0.89	1.33
C17:1*n*-9	0.72	-
C18:0, Stearic	14.35	7.26
C18:1, Oleic	1.97	-
C18:1*n*-11t	23.29	14.07
Octadecanoic acid, 17-methyl	0.18	-
C18:2*n*-6, Linoleic	2.46	-
C20:0, arachidic	0.67	0.64
C20:1, Erucic	1.88	1.04
C20:2, Eicosadienoic	1.25	-
C20:4, Eicosatetranoic	0.55	-
C21:0, Heneicosanoic	-	0.045
C22:0, Behenic	0.037	0.093
C22:4*n*-6, Adrenic	0.255	0.03
C23:0, Tricosanoic	-	0.034
C24:0, Lignoceric	0.015	0.03
C26:0, Cerotic	-	0.01
Totaal	79.317	52.346

**Table 2:** Characteristics of phytosomes containing *Callistemon citrinus* leaf extract.

Characteristic	Result (Mean ± SD)
Mean particle size (nm)	129.98 ± 18.30
Polydispersity index	1.13 ± 0.03
Entrapment efficiency (%)	80.49 ± 0.07
Temperature on the stability (106 days)	20 ± 2 °C
Total Solubility profile (%)	90.00

**Table 3:** Impact of *C. citrinus* extract and phytosomes supplementation on brain and obesity indicators in rats consuming a high-fat–fructose diet.

Measurements	C	C + V	C + C.c (200 mg/kg)	HFD	HFD + Orl (5 mg/kg)	HFD + C.c (200 mg/kg)	HFD + P (50 mg/kg)	HFD + P (100 mg/kg)	HFD + P (200 mg/kg)
Brain (g)	1.93 ± 0.03 ^a^	2.07 ± 0.02 ^a^	2.08 ± 0.07 ^a^	2.06 ± 0.04 ^a^	1.95 ± 0.05 ^a^	1.96 ± 0.04 ^a^	2.04 ± 0.08 ^a^	2.08 ± 0.03 ^a^	2.17 ± 0.02 ^a^
Body weight gain (g)	171.00 ± 36.42 ^b^	168.66 ± 9.86 ^b^	169.00 ± 35.36 ^b^	267.33 ± 36.90 ^a^	180.75 ± 40.42 ^b^	146.25 ± 31.84 ^b^	178.66 ± 27.22 ^b^	167.33 ± 23.02 ^b^	163.66 ± 3.21 ^b^
Adiposity index	2.78 ± 0.55 ^c^	2.77 ± 0.55 ^c^	2.52 ± 0.62 ^c^	9.43 ± 0.62 ^a^	5.56 ± 0.71 ^bc^	6.18 ± 0.39 ^b^	5.98 ± 0.71 ^b^	4.82 ± 0.71 ^bc^	4.02 ± 0.62 ^bc^
Lee index	0.30 ± 0.01 ^b^	0.30 ± 0.02 ^b^	0.30 ± 0.01 ^b^	0.33 ± 0.01 ^a^	0.30 ± 0.01 ^b^	0.30 ± 0.01 ^b^	0.29 ± 0.01 ^b^	0.30 ± 0.01 ^b^	0.31 ± 0.31 ^b^

All values expressed as mean ± SEM (*n =* 6; ANOVA followed by Tukey’s test, with statistically different values ^(a, b, c)^ between groups; *p* < 0.05).

**Table 4:** Percentage of the major fatty acids in the brain of control rats and the different treatments.

Fatty Acid	C	C + V	C + C.c (200 mg/kg)	HFFD	HFFD + Orl (5 mg/kg)	HFFD+ C.c (200 mg/kg)	HFFD + P (50 mg/kg)	HFFD + P (100 mg/kg)	HFFD + P (200 mg/kg)
**C14:0, Myristic**	0.12 ± 0.04 c	0.24 ± 0.04 ab	0.18 ± 0.04 abc	0.24 ± 0.03 abc	0.18 ± 0.03 abc	0.29 ± 0.08 a	0.16 ± 0.01 bc	0.17 ± 0.02 abc	0.15 ± 0.05 bc
**C15:0, Pentadecylic**	0.07 ± 0.01	0.11 ± 0.01	0.10 ± 0.01	0.10 ± 0.01	0.09 ± 0.01	0.07 ± 0.02	0.09 ± 0.01	0.07 ± 0.03	0.10 ± 0.04
**C16:0, Palmitic**	17.40 ± 1.56	20.03 ± 1.60	18.95 ± 0.91	18.80 ± 0.30	20.42 ± 1.09	18.52 ± 2.39	19.29 ± 0.66	19.18 ± 1.85	19.27 ± 1.66
**C17:0, Margaric**	0.23 ± 0.01	0.30 ± 0.02	0.27 ± 0.04	0.33 ± 0.02	0.31 ± 0.09	0.23 ± 0.03	0.29 ± 0.03	0.29 ± 0.05	0.30 ± 0.02
**C18:0, Stearic**	15.08 ± 1.85 bc	18.74 ± 0.84 ab	18.15 ± 0.67 abc	18.24 ± 0.74 abc	19.91 ± 0.95 a	14.61 ± 3.18 c	17.66 ± 0.35 abc	17.88 ± 0.60 abc	18.20 ± 0.79 abc
**C20:0, Arachidic**	0.42 ± 0.21	0.22 ± 0.08	0.26 ± 0.06	0.42 ± 0.20	0.32 ± 0.09	0.38 ± 0.16	0.23 ± 0.04	0.31 ± 0.08	0.173 ± 0.040
**C22:0, Behenic**	0.37 ± 0.20	0.29 ± 0.04	0.31 ± 0.09	0.37 ± 0.20	0.34 ± 0.14	0.32 ± 0.19	0.16 ± 0.02	0.29 ± 0.09	0.16 ± 0.05
**C16:1** ***n*** **-9, Palmitoleic**	0.41 ± 0.06 b	0.45 ± 0.05 ab	0.54± 0.02 ab	0.56 ± 0.05 a	0.49 ± 0.05 ab	0.43 ± 0.01 ab	0.50 ± 0.04 ab	0.53 ± 0.05 ab	0.49 ± 0.01 ab
**C18:1** ***n*** **-9, Oleic**	12.34 ± 0.12	14.58 ± 1.35	14.868 ±1.00	14.340 ± 1.019	16.42 ± 2.23	14.33 ± 3.51	14.263 ± 0.348	15.987 ± 0.771	15.506 ± 1.872
**C20:1** ***n*** **-9, Eicosenoic**	2.48 ± 0.06 a	0.94 ± 0.56 b	1.12 ± 0.14 b	0.95 ± 0.21 b	1.23 ± 0.41 ab	2.03 ± 0.058 ab	1.26 ± 0.36 ab	1.62 ± 0.54 ab	0.89 ± 0.25 b
**C22:1** ***n-*** **9, Erucic**	0.21 ± 0.08	0.18 ± 0.10	0.13 ± 0.06	0.14 ± 0.04	0.20 ± 0.10	0.13 ± 0.06	0.13 ± 0.05	0.20 ± 0.21	0.17 ± 0.21
**C24:1** ***n*** **-9, Nervonic**	1.33 ± 0.25 a	0.25 ± 0.18 b	0.31 ± 0.03 b	0.24 ± 0.16 b	0.55 ± 0.35 b	0.45 ± 0.07 b	0.23 ± 0.07 b	0.39 ± 0.17 b	0.21 ± 0.18 b
**C18:2** ***n*** **-6, LA**	0.81 ± 0.10 ab	0.94 ± 0.19 a	0.88 ± 0.15 ab	0.73 ± 0.10 ab	0.64 ± 0.13 ab	0.53 ± 0.12 b	0.68 ± 0.08 ab	0.68 ± 0.10 ab	0.80 ± 0.16 ab
**C20:4** ***n*** **-6. Arachidonic**	7.92 ± 1.64	9.57 ± 0.67	9.77 ± 0.31	9.44 ± 0.75	9.60 ± 1.50	7.21 ± 1.35	9.96 ± 0.14	9.91 ± 0.38	10.48 ± 0.14
**C22:4** ***n-*** **6, Adrenic**	2.08 ± 0.37 bc	2.93 ± 0.15 abc	3.18 ± 0.45 a	2.92 ± 0.33 abc	3.01 ± 0.57 ab	1.98 ± 0.46 c	3.12 ± 0.15 a	3.34 ± 0.15 a	3.15 ± 0.25 a
**C22:5** ***n*** **-3, DPAn-3**	0.25 ± 0.15 c	0.63 ± 0.18 abc	1.05 ± 0.38 ab	1.05 ± 0.30 ab	0.79 ± 0.46 abc	0.36 ± 0.09 bc	0.93 ± 0.16 abc	1.20 ± 0.17 a	1.12 ± 0.07 a
**C22:6** ***n*** **-3, DHA**	10.09 ± 2.32	11.55 ± 1.39	10.54 ± 1.10	10.35 ± 1.51	9.52 ± 3.99	6.53 ± 1.55	10.74 ± 0.77	9.95 ± 0.65	12.09 ± 0.45
**DMA C16:0**	1.66 ± 0.06	1.59 ± 0.35	1.76 ± 0.18	1.67 ± 0.43	1.79 ± 0.56	0.95 ± 0.07	1.51 ± 0.14	1.49 ± 0.45	1.68 ± 0.22
**DMA C18:0**	3.36 ± 0.37	3.48 ± 0.62	2.81 ± 1.35	3.39 ± 0.83	3.73 ± 1.08	1.87 ± 0.41	2.56 ± 0.51	2.28 ± 0.88	3.29 ± 0.17
**DMA C18:1 cis**	1.430± 0.06	0.85 ± 0.51	0.82 ± 0.40	0.91 ± 0.37	0.96 ± 0.31	0.76 ± 0.84	0.65 ± 0.13	0.88 ± 0.15	0.84 ± 0.26
**DMA C18:1 trans**	1.68 ± 0.23 a	0.88 ± 0.51 ab	0.84 ± 0.40 ab	0.87 ± 0.34 ab	0.90 ± 0.30 ab	0.77 ± 0.01 ab	0.63 ± 0.11 b	0.86 ± 0.16 ab	0.75 ± 0.24 ab

All values expressed as mean ± SEM (*n* = 6); values statistically different (a, b, c) among groups (*p* ≤ 0.05) according to Tukey’s test.

## Data Availability

The raw data supporting the conclusions of this article will be made available by the authors on request.

## Supplementary Materials

The following supporting information can be downloaded at: https://www.mdpi.com/article/10.3390/biom15081129/s1, Figure S1. GC/MS chromatogram (arbitrary units) of *C. citrinus* leaf extract illustrating its compounds. Table S1. Compounds of *Callistemon citrinus* leaves extract found in GC/MS. Table S2. Composition of standard rat chow diet and high-fat-fructose diet.
