# Supplementary material for: Effect of Callistemon citrinus Phytosomes on Oxidative Stress in the Brains of Rats Fed a High-Fat–Fructose Diet
Source: Biomolecules. 2025 Aug 5;15(8):1129. doi: 10.3390/biom15081129 (PMC12384132; doi:10.3390/biom15081129)
Supplement: Supplementary file 1 [file biomolecules-15-01129-s001.zip › Supplementary material S1.pdf]

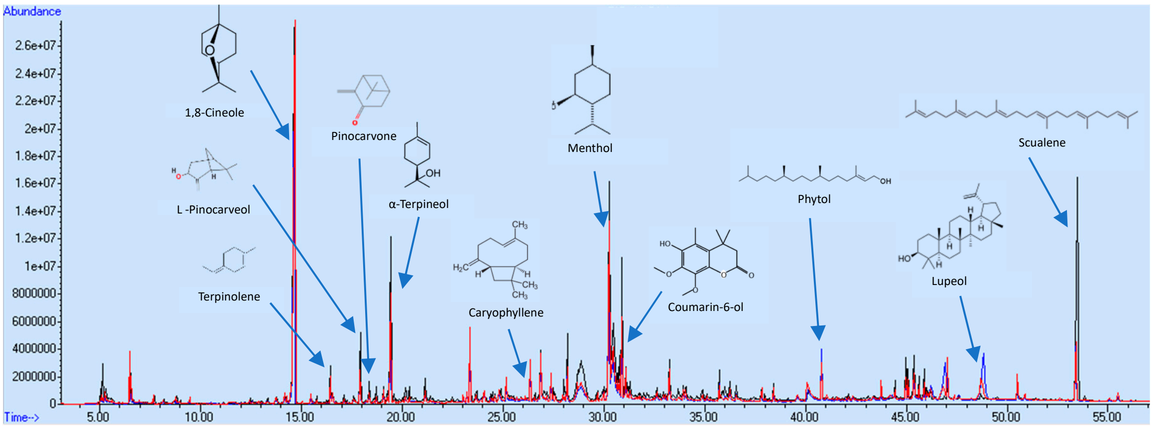

Figure S1. GC/MS chromatogram (arbitrary units) of *C. citrinus* leaf extract illustrating its compounds

Table S1. Compounds of *Callistemon citrinus* leaves extract found in GC/MS

RT	Match factor	Compounds	(%)
**14.659**	944	1,8-Cineole	14.46 ± 0.02
**16.435**	932	Terpinolene	0.68 ± .001
**17.91**	909	L-pinocarveol	1.13 ± 0.004
**18.365**	930	Pinocarvone	0.33 ± 0.001
**18.709**	939	Borneol	0.22 ± 0.001
**19.071**	940	4-Terpineol	0.35 ± 0.001
**19.271**	833	Cis-p-Menth-2,8-dienol	0.29 ± 0.001
**19.425**	940	α-Terpineol	3.55 ± 0.002
**26.352**	936	Caryophyllene	0.79 ± 0.002
**29.681**	820	Epiglobulol	0.25 ± 0.001
**30.255**	788	Menthol	6.47 ± 0.001
**30.886**	649	Coumarin-6-ol, 3,4-dihydro-7,8-dimethoxy-4,4,5-trimethyl-	2.39 ± 0.004
**40.804**	943	Phytol	1.06 ± 0.004
**46.213**	773	α-Amyrin	0.50 ± 0.003
**48.842**	920	Lupeol	3.97 ± 0.024
**53.408**	924	Squalene	4.31 ± 0.037
