# Supplementary material for: Effect of Callistemon citrinus Phytosomes on Oxidative Stress in the Brains of Rats Fed a High-Fat–Fructose Diet
Source: Biomolecules. 2025 Aug 5;15(8):1129. doi: 10.3390/biom15081129 (PMC12384132; doi:10.3390/biom15081129)
Supplement: Supplementary file 1 [file biomolecules-15-01129-s001.zip › Supplementary material S2.pdf]

Table S2. Composition of standard rat chow diet and high-fat-fructose diet

Ingredients	standard (g/100g)	High-Fat-Fructose diet (g/100g)
Powdered rat feed	0	45.4
Pork lard	0	14.8
Vegetal shortening	0	14.8
Fructose	0	25.0
Calcium	0.8	0.8
Phosphorus	0.4	0.4
Fiber	5	5
Moisture	13	13
Ash	8	8
Nutrients	%/100g	%/100g
Carbohydrate	48.8	29.8
Protein	21	28.5
Fat	3	41.6
Mineral mix	35	35
Vitamin	10	10
Energy content	3.06 kcal/g	6.08 kcal/g
